# CRISPR-dependent base editing as a therapeutic strategy for rare monogenic disorders

**DOI:** 10.3389/fgeed.2025.1553590

**Published:** 2025-04-02

**Authors:** Júlia-Jié Cabré-Romans, Raquel Cuella-Martin

**Affiliations:** ^1^ Department of Human Genetics, McGill University, Montreal, QC, Canada; ^2^ Victor Phillip Dahdaleh Institute of Genomic Medicine, McGill University, Montreal, QC, Canada

**Keywords:** gene therapy, base editing, rare monogenic disease, CRISPR-based therapeutics, mutation correction

## Abstract

Rare monogenic disorders are caused by mutations in single genes and have an incidence rate of less than 0.5%. Due to their low prevalence, these diseases often attract limited research and commercial interest, leading to significant unmet medical needs. In a therapeutic landscape where treatments are targeted to manage symptoms, gene editing therapy emerges as a promising approach to craft curative and lasting treatments for these patients, often referred to as “one-and-done” therapeutics. CRISPR-dependent base editing enables the precise correction of genetic mutations by direct modification of DNA bases without creating potentially deleterious DNA double-strand breaks. Base editors combine a nickase version of Cas9 with cytosine or adenine deaminases to convert C·G to T·A and A·T to G·C, respectively. Together, cytosine (CBE) and adenine (ABE) base editors can theoretically correct ∼95% of pathogenic transition mutations cataloged in ClinVar. This mini-review explores the application of base editing as a therapeutic approach for rare monogenic disorders. It provides an overview of the state of gene therapies and a comprehensive compilation of preclinical studies using base editing to treat rare monogenic disorders. Key considerations for designing base editing-driven therapeutics are summarized in a user-friendly guide for researchers interested in applying this technology to a specific rare monogenic disorder. Finally, we discuss the prospects and challenges for bench-to-bedside translation of base editing therapies for rare monogenic disorders.

## 1 Introduction

The term “rare disorders” encompasses a group of ∼7,000 human diseases affecting less than one in 2,000 people in any given World Health Organization region (Wang et al., 2021; The Lancet Global, 2024; National Institutes of Health, 2023). The worldwide burden is significant: Approximately 300 million people live with rare diseases, 70% of which present in childhood, and such individuals are often neglected and marginalized, especially in low- and middle-income countries (The Lancet Global, 2024).

Around 80% of rare disorders are caused by a single-gene defect, *i.e.*, monogenic (National Institutes of Health, 2023). Despite knowledge of their molecular causes, the low prevalence of rare disorders makes them difficult to recognize, accurately diagnose, and treat, attracting limited research and commercial interest (Zhang and Wu, 2024). The average time for an accurate diagnosis is 4–8 years, and about 30% of children with a rare disease die before the age of five (The Lancet Global, 2024). The limited availability of animal models and clinical specimens has further constrained preclinical studies and translation of therapies for rare diseases, creating unmet medical needs (Hmeljak and Justice, 2019). As of today, only 500 rare disorders count with available treatments, focusing primarily on symptom management with few curative options (The Lancet Global, 2024; National Institutes of Health, 2023).

Gene therapy has emerged as a promising approach to craft curative treatments for these patients (Kirschner and Cathomen, 2020). Gene therapy modifies a person’s genetic material to treat or prevent a disease (FDA, 2018). This umbrella term includes strategies like gene replacement, gene silencing, and gene editing (Zhang and Wu, 2024). While gene replacement and silencing strategies can compensate for protein loss-of-function or inhibit gain-of-function mutation, overexpressing or silencing a gene may be insufficient to correct a specific disease phenotype (Raguram et al., 2022). Gene editing therapies, including CRISPR-dependent base editing, can treat a broader range of genetic diseases, as they can directly correct pathogenic mutations in the genomic DNA. Achieving the same effects as gene replacement/silencing strategies, corrective gene editing approaches offer the potential for long-lasting effects in the form of ‘one-and-done’ treatments (Raguram et al., 2022).

Here, we provide a user-guide-like review of CRISPR-dependent base editing applications for treating rare monogenic disorders. We discuss current research on base editing as a therapeutic option, including recent advances and potential challenges to provide a reference for future research in this rapidly evolving field. To conclude, we elaborate on the outlook of these therapies and highlight potential actions to streamline bench-to-bedside translation.

## 2 Gene therapies for rare monogenic disorders: the current landscape

Gene therapies aim to directly correct or modify the genetic cause of a disease. Gene therapies offer the potential for personalized treatments with reduced side effects, leading the transition from symptom management to curative treatments for rare disorders. Gene therapy can be categorized into three distinct approaches: gene replacement, silencing, and correction, each with unique mechanisms and potential advantages (Yan et al., 2024).

Gene replacement strategies deliver an exogenous functional complementary DNA copy of a defective gene into the patient’s living cells to restore normal cellular function. This approach is a good candidate for diseases caused by mutations resulting in a specific protein’s absence or malfunction advantages (Yan et al., 2024; Petrich et al., 2020). For example, spinal muscular atrophy (SMA) can be treated using adeno-associated virus (AAV)-mediated gene replacement to express the functional *SMN1* gene, as demonstrated by the therapeutic onasemnogene abeparvovec-xioi (Alves et al., 2024).

Gene silencing uses small RNA or DNA molecules, i.e., antisense oligonucleotides (ASOs) or small interference RNA (siRNA), to inhibit the expression of specific genes and reduce the production of a disease-causing protein. ASO and siRNA therapies target the mRNA; they do not alter the patient’s DNA, leaving the pathogenic variant uncorrected and requiring lifelong intermittent administration (Alves et al., 2024). ASOs are short oligonucleotides that bind to RNA in a target-specific manner. Their mechanisms of action involve knocking down mRNAs but also modifying pre-mRNA splicing, leading to a reduction, modification, or restoration of a specific protein (Lauffer et al., 2024). They are extremely versatile molecules and can be tailored for individual cases for disorders with only a few known cases, critical to treat rare disorders (Kim et al., 2023; Kim et al., 2019). The Food Drug Administration (FDA) has approved a total of twelve ASO drugs (Vinjamuri et al., 2024), some of which treat rare diseases like Duchenne muscular dystrophy (DMD) and SMA (Alves et al., 2024; Duan et al., 2021). siRNAs are double-stranded RNA nucleotide sequences that bind to and degrade mRNA silencing gene expression (Collotta et al., 2023). To date, the FDA has approved six siRNA-based drugs, including patisiran and lumasiran that treat the rare diseases acute hepatic porphyria and primary hyperoxaluria type 1, respectively (Padda et al., 2025).

By using CRISPR/Cas technologies like CRISPR-Cas9 or CRISPR-dependent base editors, gene editing therapy enables precise modifications to the patient’s DNA to delete, replace, or insert genetic material. Noteworthily, in late 2023, exagamglogene autotemcel (Casgevy^®^) was the first FDA-approved cell therapy utilizing *ex-vivo* CRISPR/Cas9 technology for the treatment of sickle cell disease, a rare inherited blood disorder (Frangoul et al., 2024). Although CRISPR-based gene therapies have not yet received full regulatory approval, clinical trials are underway for diseases including neurological disorders and hypercholesterolemia (Deneault, 2024).

## 3 CRISPR-dependent base editing: from DNA cutting to direct mutation insertion

CRISPR-based systems are composed of a Cas protein with endonuclease activity and a single guide RNA (sgRNA) that directs the ribonucleoprotein complex to the desired site of the genome (Gaudelli et al., 2017). A protospacer-adjacent motif (PAM) sequence near the target site is required for effective DNA targeting (Hille et al., 2018). Recognition of the target site begins when the Cas-sgRNA complex binds to the matching PAM sequence unwinding the double-stranded DNA (Jiang and Doudna, 2017; Sternberg et al., 2014). The sgRNA protospacer creates an R-loop that exposes the non-target DNA strand, making it accessible to other molecules. In traditional CRISPR-Cas9 systems, Cas9 then undergoes conformational changes that activate its nuclease domains and produce a DNA double-strand break (DSB). These structural changes are hindered by mismatches between the target DNA strand and the sgRNA protospacer, limiting nuclease activation to sequences highly complementary to the sgRNA protospacer (Anzalone et al., 2020). Differentiated mammalian cells largely repair nuclease-induced DSBs through non-homologous end-joining, directly ligating DNA ends, often resulting in nucleotide insertion and deletions around the break site that abrogate gene function (Anzalone et al., 2020; Mali et al., 2013; Xu and Li, 2020; Doench et al., 2014).

While CRISPR-Cas9 is very efficient at selectively disrupting target gene sequences (Mali et al., 2013; Cong et al., 2013), CRISPR-dependent base editing can directly correct genetic mutations by precise modification of DNA bases (Komor et al., 2016). Base editors combine an engineered Cas9 that inserts single-strand DNA breaks (nickase) fused to cytosine or adenine deaminases. Base editor-mediated mutations occur within a nucleotide activity window defined by the sgRNA, corresponding to the nucleotides in the R-loop that interact with the deaminase (Jiang and Doudna, 2017; Nishimasu et al., 2014). The activity window can be additionally influenced by differences in DNA state, such as chromatin architecture, that can vary by locus or cell type (Gaudelli et al., 2017; Komor et al., 2016). Unlike traditional CRISPR-Cas9 technologies, base editor-induced modifications do not require DSB generation, thus avoiding potential indels at the target locus, chromosomal rearrangements, and p53-driven stress responses (Komor et al., 2016).

Cytosine base editors (CBEs) employ a cytosine deaminase converting cytosines within the R-loop into uracils, which polymerases interpret as thymines (Komor et al., 2016; Komor et al., 2017). CBEs use APOBEC1 deaminase, with later versions incorporating other APOBEC family members (A3A-D, A3F-H) and other deaminases such as CDA or AID with varying kinetic parameters, nucleotide substrate preference, and editing window widths (Ma et al., 2016; Yu et al., 2020; Wu et al., 2019; Yuan et al., 2018). Cytosine deamination to uridine creates a DNA base pair mismatch from the non-deaminated strand that, upon DNA replication, results in a base replacement of the unedited strand. However, uracil is mutagenic and is often rapidly excised by uracil DNA glycosylase (UNG) (Wood, 1996; Radany et al., 2000); CBEs typically incorporate UNG inhibitor proteins (UGIs) to enhance editing efficiency by preventing reversion of the modified base (Komor et al., 2017; Wang et al., 2017). Interestingly, base transversion has been achieved through the fusion of CBEs to UNG (CGBEs) (Wang et al., 2024). UNG excises the deaminated base to generate apurinic/apyrimidinic sites that are repaired by base excision or translesion synthesis leading to a versatile nucleotide replacement (Wang et al., 2024). CGBEs incorporate C-to-A transversions in *E. coli* and C-to-G transversions in mammalian cells (Zhao et al., 2021).

Adenine base editors (ABEs) use a laboratory-evolved mutant of the TadA tRNA deoxyadenosine deaminase (TadA*) that converts adenines within the DNA R-loop to inosines, which are read as guanines by DNA polymerases (Gaudelli et al., 2017). Inosine excision in mammalian cells is less efficient than uracil excision as inhibiting MPG—the glycosylase believed to remove inosine from genomic DNA in eukaryotic cells—failed to enhance editing product purities (Gaudelli et al., 2017). TadA* editing efficiency and Cas9 compatibility were significantly improved by phage-assisted evolution, crafting the ABE8 generation, go-to editors in molecular biology laboratories and therapeutic applications alike (Gaudelli et al., 2017).

## 4 Testing base editing for rare monogenic disorders: what to consider?

CRISPR-dependent base editors have already been successfully applied in preclinical *in vivo* research for more than twenty-five disorders, including more than ten rare monogenic disorders, compiled in detail in Table 1 (Zhao et al., 2021).

**TABLE 1:** *In vivo* gene editing strategies used in preclinical studies for rare monogenic disorders.

DiseaseDisease	Prevalence	Model	Key organ (cell type)	Targeted gene	Editing strategy	Editing outcome	Editor variant	Delivery method	Administration	Ref
Phenylketonuria	1/10,000	Mice	Liver (hepatocytes)	*Pah* c.835 T>C (p.F263S)	Direct mutation correction	• 10% editing after 4 weeks increasing to 25% after 26 weeks • Return of blood phenylalanine to normal levels	SaCas9KKH-BE3	Intein-based dual AAV8	Systemic (tail vein injection)	Villiger et al. (2018)
		Mice	Liver (hepatocytes)	*Pah* c.835 T>C (p.F263S)	Direct mutation correction	• 23% editing after 8 weeks using AAV • 19% editing 1 week after second LNP dose • Return of blood phenylalanine to normal levels	SaCas9KKH-BE3	Intein-based dual AAV8 and LNP encapsulating mRNA	Systemic (tail vein injection)	Villiger et al. (2021)
		Mice	Liver (hepatocytes)	*Pah* c.835T>C (p.F263S)	Direct mutation correction	• 6% editing and 32% reduced blood phenylalanine	SaCas9KKH-BE3	Intein-based dual AAV8	Systemic (facial vein injection)	Zhou et al. (2022)
		Humanized mice	Liver (hepatocytes)	*PAH* c.1222C>T (p.R408W)	Direct mutation correction	• 26% on-target editing, with 4% bystander editing	ABE8.8-SpRY	LNP encapsulating mRNA	Systemic (retro-orbital injection)	Brooks et al. (2024)
		Humanized mice	Liver (hepatocytes)	*PAH* c.1222C>T (p.R408W)	Direct mutation correction	• 19.1% to 34.6% editing, with 1.4% bystander editing	ABE8e-SpRY	Intein-based dual AAV8	Systemic (tail vein injection)	Yin et al. (2025)
Hereditary tyrosinemia type I	1/100,000	Mice	Liver (hepatocytes)	*Fah* G>A in last nucleotide of exon 8	Direct mutation correction	• 9.5% editing and restoration of mouse body weight	optimized ABE6.3	Hydrodynamic injection of DNA	Systemic (tail vein injection)	Song et al. (2020)
		Mice	Liver (hepatocytes)	*Fah* G>A in last nucleotide of exon 8	Direct mutation correction	• 12.5% editing and restoration of mouse body weight	optimized ABE6.3	LNP encapsulating mRNA	Systemic (tail vein injection)	Jiang et al. (2020)
		Mice	Liver (hepatocytes)	*Fah* G>A in last nucleotide of exon 8	Direct mutation correction	• 58.1% editing in liver tissues with minimal bystander editing.	haA3A-CBE	Intein-based dual AAV8, and LNP encapsulating mRNA	Systemic (tail vein injection)	Yang et al. (2024)
Alpha-1 antitrypsin deficiency	1-5/10,000	Mice	Liver (hepatocytes)	*PiZ SERPINA1* c.1096G>A (p.E342K)	Install a compensatory mutation (p.M374I) that stabilizes AAT protein	• 28.5% compensatory editing in the liver after 1 week, 34.3% after 12 weeks, and 27.2% after 32 weeks	BE4	LNP encapsulating mRNA	Systemic (tail vein injection)	Packer et al. (2022)
					Direct mutation correction	• 12% editing rate in the liver after 1 week, 29% after 12 weeks, and 35.7% after 32 weeks	ngcABEvar9			
Hutchinson-Gilford progeria syndrome	<1/1,000,000	Humanized mice	Heart (vascular smooth muscle cells)	*LMNA* c.1824C > T (p.G608G)	Direct mutation correction	• 30% editing in heart, 20% in aorta • 2.4-fold increase in lifespan	ABE7.10max-VRQR	Intein-based dual AAV9	Systemic (retro-orbital or intraperitoneal injection)	Koblan et al. (2021)
		Humanized mice	Skin cells	*LMNA* c.1824C > T (p.G608G)	Direct mutation correction	• 20.8%–24.1% editing in mice skin cells	ABEmax-VQR	Transient non-integrative MS2-lentiviral particle vector system (LentiFlash®)	Systemic (intraperitoneal injection)	Whisenant et al. (2022)
Duchenne muscular dystrophy	1-9/100,000	Mice	Skeletal muscle (myofibers)	*Dmd* nonsense mutation Q>X in exon 20	Direct mutation correction	• 3.3% local editing • 17% of local myofibers stained for restored dystrophin	ABE7.10	Dual trans-splicing AAV9	Localized (intramuscular injection)	Ryu et al. (2018)
		Mice	Skeletal muscle (myofibers)	*Dmd* exon 51 deletion	Direct mutation correction	• 35% local editing • 96% of local myofibers stained for restored dystrophin	ABE7.10max	Intein-based dual AAV9	Localized (intramuscular injection in the tibialis anterior muscle)	Chemello et al. (2021)
		Humanized mice	Skeletal muscle (myofibers)	*DMD* c.4174C>T, (p.Q1392X)	Direct mutation correction	• 35% editing with 54% dystrophin restoration	RNA base editor mxABE	Single AAV9	Localized (intramuscular injection)	Li et al. (2023)
		Humanized mice	Skeletal muscle (myofibers)	*DMD* exon 51 deletion	Mutation insertion to disrupt DMD splicing sites	• ∼15% editing resulting in over 70% exon skipping efficiency • Up to 96% of local myofibers stained for restored dystrophin	ABE8e	Intein-based dual AAV	Localized (intramuscular injection)	Lin et al. (2024)
Spinal muscular atrophy	0.85/100,000	Mice	Brain and spinal cord cells	*Smn2* 6th nucleotide of exon 7	Compensatory base editing of *Smn2* T6>C, a paralogous gene to *Smn1* to restore Smn protein levels	• 87% average T6>C conversion, improved motor function, and extended average lifespan	ABE8e-SpyMac	Intein-based dual AAV9	Localized (intracerebroventricular injection)	Arbab et al. (2023)
		Mice	Brain and spinal cord cells	*Smn2* 6th nucleotide of exon 7	Compensatory base editing of *Smn2* T6>C, a paralogous gene to *Smn1* to restore Smn protein levels	• ∼6% editing in brain and ∼4% in spinal cord at day 13 • ∼10% editing in brain and ∼8% in spinal cord at week 12	ABE8e-SpRY	Intein-based dual AAV9	Localized (intracerebroventricular injection) and Systemic (retroorbital injection)	Alves et al. (2024)
Sickle cell disease/ β-thalassemia	1–5/10,000	Humanized mice	Blood (hematopoietic stem cells)	*Hpfh* c.*113A>G	Direct mutation correction	• 30% editing following selection • 21% of blood β-like globins were fetal hemoglobin	ABE7.10max	Adenovirus	Systemic (intravenous)	Li et al. (2021)
		Humanized mice	Blood (hematopoietic stem cells)	*Hpfh* c.*113A>G	Direct mutation correction	• 60% editing with 30% γ-globin of β-globin expressed in 70% of erythrocytes	ABE8e	Adenovirus	Systemic (intravenous)	Li et al. (2022)
Amyotrophic lateral sclerosis	1-9/100,000	Humanized mice	Central nervous system (motor neurons)	*Sod1* c.281G>C (p.G93A)	Direct mutation correction	• 1.2% editing • 11% increase in lifetime, • 85% increase in duration between onset of late-stage disease and death	BE3	Intein-based dual AAV9	Localized (lumbar subarachnoid space injection)	Lim et al. (2020)
Niemann-Pick disease type C	1-9/100,000	Mice	Central nervous system (Purkinje cells)	*Npc1* c.3182T>C (p.I1061T)	Direct mutation correction	• 48% editing in cortex, 42% in Purkinje cells, up to 59% in cortex at test site • 10% increase in lifetime	BE3.9max	Intein-based dual AAV9	Systemic (retro-orbital injection)	Levy et al. (2020)
Hurler syndrome	1-9/1,000,000	Mice	Liver and heart (progenitor cells)	*Idua* G>A (p.W392X)	Direct mutation correction	• ∼12.8% editing in liver progenitor cells, ∼12.6% in myocytes, ∼3.0% in endothelial cells, ∼2.3% in fibroblasts	ABE7.10max	*In utero* intein-based dual AAV9	Systemic	Bose et al. (2021)
		Mice	Liver, heart, brain (hepatocytes, cardiomyocites and neurones)	*Idua* G>A (p.W392X)	Direct mutation correction	• ∼22.46% editing in hepatocytes, 11.18% in heart, and 0.12% in the brain	ABE8e-SpG	Intein-based dual AAV9	Systemic (temporal vein injection)	Su et al. (2023)
		Mice	Brain (midbrain, hippocampus, forebrain)	*Idua* G>A (p.W392X)	Direct mutation correction	• ∼1% editing in midbrain, hippocampus, and forebrain	ABE7.10	C3-LNP encapsulating mRNA	Localized (intracerebroventricular)	Palanki et al. (2023)
Leber congenital amaurosis	1-9/100,000	Mice	Retina (retinal pigmented epithelium)	*Rpe65* c.130C>T (p.R44X)	Direct mutation correction	• 15% editing, restored visual function.	ABE7.10max	Lentivirus	Localized (subretinal injection)	Suh et al. (2021)
		Mice	Retina (retinal pigmented epithelium)	*Rpe65* c.130C>T (p.R44X)	Direct mutation correction	• 14% editing, restored visual function • 89% editing in *Rpe65* cDNA	ABE7.10max-SpCas9-NG	Intein-based dual AAV9	Localized (subretinal injection)	Jo et al. (2023)
		Mice	Retina (retinal pigmented epithelium)	*Rpe65* c.130C>T (p.R44X)	Direct mutation correction	• 54% editing with 40% of visual function restored	NG-ABE	Lentivirus	Localized (subretinal injection)	Choi et al. (2022)
		Mice	Retina (retinal pigmented epithelium)	*Kcnj13* c.158G>A (p.W53X)	Direct mutation correction	• ∼20% editing in retinal pigmented epithelial cells	ABE8e	Silica nanocapsules encapsulating mRNA	Localized (subretinal injection)	Kabra et al. (2023)
		Mice	Retina (retinal pigmented epithelium)	*Rpe65* c.130C>T (p.R44X)	Direct mutation correction	• 16.38% editing with ABE8e • 11.33% editing with ABEmax • 9.96% editing with ABE8e-WQ	ABE8e, ABEmax, ABE8e-WQ	Intein-based dual AAV2/9	Localized (subretinal injection)	Lee et al. (2024)
Recessive hearing loss		Mice	Inner ear (hair cells)	*Tmc1* c.545A>G (p.Y182C)	Direct mutation correction	• 2.3% bulk genomic correction, 33% cDNA correction • Increased hearing at 4 weeks that slowly degenerated	AID-BE3.9max	AAV (Anc80 serotype)	Localized (inner-ear injection)	Yeh et al. (2020)
	3% of prelingual deafness in Spain	Humanized mice	Inner ear (hair cells)	*OTOF* c.2485C>T (p.Q829X)	Direct mutation correction	• 80% editing • ∼100% OTOF protein restoration of inner hair cells	RNA base editor emxABE	AAV9	Localized (inner-ear injection)	Xue et al. (2023)
Long QT syndrome type 3	1–5/10,000	Mice	Heart (cardiomyocytes)	*Scn5a* c.3908 C>T (p.T1307M)	Direct mutation correction	• ∼40% editing • Up to 99% restored *Scn5a* mRNA levels and restored mouse QT and QTc phenotype	ABEmax	Intein-based dual AAV9	Systemic (intraperitoneal injection)	Qi et al. (2024)

Of note, the progress of base editing technologies in gene therapeutics has already extended beyond preclinical applications. Clinical trials are underway for therapeutics for the common monogenic disorder heterozygous familial hypercholesterolemia (VERVE-101/102) and the rare monogenic disorder alpha-1 antitrypsin deficiency (BEAM-301), with initial results from VERVE-101 showing promising efficacy profiles (Horie and Ono, 2024; Arnaoutova et al., 2024).

**FIGURE 1:**
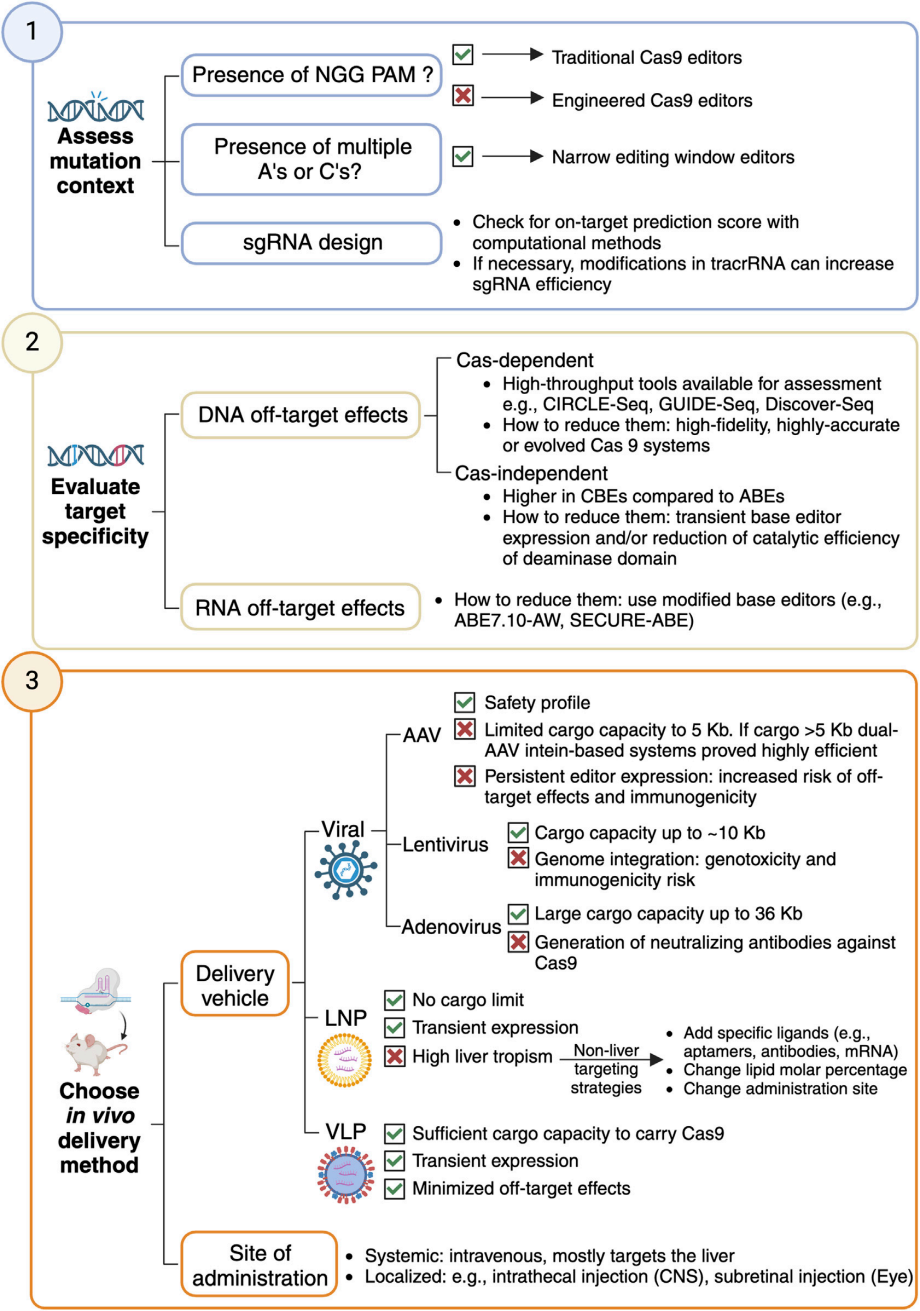
Workflow to design a base editing strategy for gene therapeutics for a rare monogenic disorder.

Can your rare monogenic disorder of interest disease be targeted with base editing-driven gene therapeutics? Here are some key considerations to explore the application of base editing in preclinical research (Figure 1).

### 4.1 Sequence context: PAM availability, editing window, and sgRNA design

The availability of a compatible PAM sequence is a key factor in determining whether a target can be edited with a base editor. The initial CBE and ABE variants were developed using SpCas9, which requires an NGG PAM. NGG-targeting CBEs can potentially correct ∼26% of annotated pathogenic T·A-to-C·G mutations in ClinVar, while NGG-targeting ABEs could theoretically correct 28% of pathogenic G·C-to-A·T point mutations (Gaudelli et al., 2017; Komor et al., 2016; Landrum et al., 2016; Hu et al., 2018). Engineered Cas9 variants have been developed to recognize alternative PAM sequences and expand the targeting scope of base editors. The VQR, EQR, and VRER SpCas9 variants changed the PAM specificity from NGG to NGA, NGAG or NGCG, respectively (Kleinstiver et al., 2015). Non-G SpCas9s, including SpCas9-NRRH, SpCas9-NRTH and SpCas9-NRCH, collectively recognized NRNH PAMs (where R is A or G and H is A, C or T) (Miller et al., 2020). Cas9-NG/SpG relaxed the PAM restriction from NGG to NGN (Kleinstiver et al., 2015; Miller et al., 2020). SpRY, a near PAM-less SpCas9, has optimal activity on NRN PAMs and low activity over NYN PAMs (where Y is C or T) (Nishimasu et al., 2018; Walton et al., 2020). Base editors have also been assembled with Cas homologs such as SaCas9 versions and Cas12a, further expanding their targeting scope (Kleinstiver et al., 2019; Li et al., 2018; Kim et al., 2017). Together, these variants will theoretically enable CBEs and ABEs to revert ∼95% of pathogenic transition mutations in ClinVar (Landrum et al., 2016).

The window of activity of base editors coincides with the R-loop nucleotides in contact with the deaminase and ranges from 5 to 10 nucleotides (Zhang and Wu, 2024). This window width may not be suitable for correcting point mutations in sequence contexts with multiple As or Cs. Mutations engineered to limit deaminase-R-loop contacts have generated editors with narrower windows of activity (e.g., F148A in TadA, and W90Y, R126E, R132E in rat APOBEC1), limiting the insertion of undesired bystander mutations (Zhou et al., 2019; Liu et al., 2020; Doman et al., 2020).

Straightforward sgRNA design can be achieved using web tools that compile information on predicted Cas9 efficiency and specificity (Konstantakos et al., 2022). Users can opt for widely used tools largely designed for knockout approaches (e.g., CRISPick, CRISPOR, and CHOPCHOP) (Doench et al., 2016; Sanson et al., 2018; Concordet and Haeussler, 2018), or more specific tools such as BE-designer, tailored to base editing experiments (Hwang et al., 2018). Computational methods have analyzed large-scale CRISPR screens to identify sgRNA and locus-features modulating Cas9 activity (Konstantakos et al., 2022): On-target prediction scores designed for Cas9 are a good surrogate measure for base editing efficiency (Cuella-Martin et al., 2021; Hanna et al., 2021). In addition, machine learning methods trained in base editing screening data, such as BeHive (Arbab et al., 2020), can provide fine-grained efficiency and editing outcome information for specific base editor-sgRNA pairs. Finally, modification in the tracrRNAs, the part of the sgRNA acting as a scaffold and binding to Cas9, may be considered to increase sgRNA efficiency. Adding mutations to disrupt a natural poly-T tract in the tracrRNA can prevent premature transcription termination resulting in better editing efficiency (Scott et al., 2019; DeWeirdt et al., 2022).

### 4.2 Target specificity

A major concern for CRISPR-dependent base editing therapies is undesired, off-target editing, both at DNA and RNA sites. Off-target DNA base editing can occur in a Cas-dependent (guided) or a Cas-independent (unguided) manner.

#### 4.2.1 Cas-dependent DNA off-target editing

The Cas9-sgRNA complex may bind to sequences sharing similarity with the target site. A productive off-target base editing requires additional criteria, such as the presence of a targetable base within the editing window and nucleotide sequence context, that are not satisfied for all Cas nuclease-dependent off-target sites (Anzalone et al., 2020). Modification of Cas9-DNA contacts and Cas9 conformation using rational design and systematic screening resulted in high-fidelity (HF), highly accurate (Hypa) and evolved (Evo) systems that minimize Cas-dependent off-target effects (Anzalone et al., 2020; Skeens et al., 2024). High-throughput techniques like CIRCLE-Seq, Discover-Seq, and Guide-Seq can systematically identify candidate loci subject to Cas-dependent off-target base editing; potential off-target sites can then be validated with an orthogonal approach (e.g., amplicon sequencing) *in cellula* or *in vivo* (Wienert et al., 2019; Tsai et al., 2017; Tsai et al., 2015). Of note, the off-target profiles of base editors may differ from that of Cas9 due to additional requirements for productive base editing (e.g., presence of a targetable base in the editing window), prompting the development of high-throughput technologies to specifically detect base editing off-targets, such as CHANGE-seq-BE (Lazzarotto et al., 2024).

#### 4.2.2 Cas-independent DNA off-target editing

Long-term deaminase expression can result in random deamination of nucleotides transiently accessible across the genome (Jin et al., 2019; Lee et al., 2020; Zuo et al., 2019). This type of off-target editing has been observed in mammalian and plant cells expressing CBEs but not in ABEs (Jin et al., 2019; Lee et al., 2020; Zuo et al., 2019). These edits occur at low frequency, with ∼5 × 10^−8^ mutations per base pair in mouse embryos injected with high levels of CBE mRNA, which is below most reported rates of spontaneous somatic cell mutation (Zuo et al., 2019). Using delivery strategies that achieve transient base editor expression limits off-target effects while preserving on-target editing due to a quicker action of BEs at on-target rather than off-target loci (Rees et al., 2017). As an alternative strategy, introducing mutations to the deaminase domain to reduce its catalytic efficiency will slow the kinetics rate at off-target *vs* on-target sites (Liu et al., 2020).

#### 4.2.3 RNA off-target editing

Most widely used deaminase domains in CBEs and ABEs are derived from enzymes that natively deaminate RNA, which can drive RNA off-target base editing when overexpressed (Richter et al., 2020; Rees et al., 2019). This phenomenon occurs at low levels in a random and widespread manner similar to Cas-independent DNA off-target editing. For instance, transient ABE7.10 overexpression in HEK293T cells has been observed to induce ∼0.22–0.24% A-to-I deamination across the transcriptome, compared with ∼0.14–0.19% A-to-I deamination from endogenous cellular adenosine deaminases (Richter et al., 2020; Rees et al., 2019). RNA off-target editing activity has been mitigated by the development of the ABE7.10-AW (Rees et al., 2019) and SECURE-ABE editors (Grünewald et al., 2019), and the introduction of V106W mutation in both ABE8 and ABE8e showed reduction RNA editing levels similar to those from cellular RNA deamination (Richter et al., 2020).

### 4.3 Delivery methods

Just like other gene therapy methods, base editor systems rely on a vehicle to reach their target cells (Zhang and Wu, 2024). Gene-editing agents can be delivered as DNA, mRNA or ribonucleoproteins (RNPs), that can be packaged into different vehicles to overcome biological barriers (Raguram et al., 2022), namely,

#### 4.3.1 Viral-based vehicles

Most *in vivo* gene editing applications use AAVs as delivery vehicles, and, to a lesser extent, lentiviruses and adenoviruses (Raguram et al., 2022; Eichler et al., 2024).

##### 4.3.1.1 Adeno-associated virus (AAV)

AAVs are widely used due to their safety profile and the characterized tissue tropism for different serotypes (Costa Verdera et al., 2020) though their limited packaging capacity (5 Kb) restricts delivery of full base editor constructs. To overcome this limitation, dual-AAV strategies, such as intein-mediated protein reconstitution, have been developed (Chemello et al., 2021; Chen et al., 2020), achieving editing efficiencies from 9% to 60% across different therapeutic organs such as liver, eye, and cardiac or skeletal muscle (Alves et al., 2024; Koblan et al., 2021; Levy et al., 2020). Intein-mediated dual-AAV deliveries were successful at introducing ABEs in progeria and SMA mouse models (Alves et al., 2024; Koblan et al., 2021). However, AAV-based delivery results in persistent Cas9 expression, risking off-target effects and immune responses that may lead to edited cell destruction (Raguram et al., 2022). Solutions include self-inactivating AAV systems and conditional base editor activation restricted to on-target loci sites (Ibraheim et al., 2021). AAVs have been a successful delivery system in preclinical research of base editing therapies against DMD (Chemello et al., 2021), amyotrophic lateral sclerosis (Lim et al., 2020), and Hutchingson-Gilford progeria syndrome (Koblan et al., 2021), among others.

##### 4.3.1.2 Lentivirus

Lentiviruses offer larger cargo capacity (∼10 Kb), and support multiplex genome editing using CRISPR-based agents but will integrate into the genome, raising risks of genotoxicity and immunogenicity that may limit their clinical potential (Milone and O’Doherty, 2018; Kymäläinen et al., 2014). *In vivo* studies have demonstrated lentiviral efficacy: An ABE delivered to the retina of a mouse model of Leber congenital amaurosis achieved 15% mutation reversion in the *Rpe65* gene and restored visual function (Suh et al., 2021).

##### 4.3.1.3 Adenovirus

Adenoviruses allow large cargoes (up to 36 Kb) and are genetically stable, with well-characterized biology and scalable production (Lee et al., 2017). CRISPR/Cas systems are commonly delivered by adenoviruses, a strategy recently used to deliver an ABE to achieve direct correction of the *Hpfh* c.*113A>G mutation responsible for beta-thalassemia (Li et al., 2021). Despite their proven efficiency for *in vivo* editing, adenoviruses may lead to the generation of neutralizing antibodies against Cas9, potentially due to the immunogenic nature of the vector (Wang et al., 2015).

#### 4.3.2 Non-viral-based vehicles

Although viral vectors have been widely used in clinical trials to deliver CRISPR/Cas systems, the immune response triggered by the vector and/or the CRISPR/Cas components remains a significant concern since it could lead to severe or even deadly side effects (Song et al., 2024). To address the limitations of viral vectors, non-viral alternatives have been developed to deliver CRISPR/Cas machineries.

##### 4.3.2.1 Lipid nanoparticles (LNPs)

LNPs, a fully synthetic option, are FDA-approved for delivery of siRNA and mRNA therapeutics. They provide transient expression with lower immunogenicity than viral vectors and can support repeated dosing (Kenjo et al., 2021). LNP-based ABE delivery showed promising results in disrupting *PCSK9* in the liver with minimal off-target effects, resulting in durable LDL-cholesterol reduction in mice and primate models of hypercholesterolemia (Musunuru et al., 2021; Rothgangl et al., 2021). However, the progress of this therapy to clinical trials highlighted the need for LNP optimization to prevent potential LNP-associated side effects: Despite the high efficiency of the treatment in patients, potential LNP-associated liver abnormalities were observed (Philippidis, 2024). In this respect, base editing therapies can benefit from research on delivery methods for gene therapeutics, such as using sugars like N-acetylgalactosamine (GalNac) to enhance overall drug uptake and delivery. GalNacs are used in FDA-approved gene therapies like eplontersen, givosiran or lumasiran and showed good tolerance and liver targeting using either the asialoglycoprotein or the low-density lipoprotein receptors. GalNac-LNPs are currently undergoing clinical trials for ABE-based treatment of hypercholesterolemia (VERVE-102) (Vafai et al., 2024). An LNP-based base editing therapy is also in clinical trials for alpha-1 antitrypsin deficiency (BEAM-302), highlighting this vehicle as the go-to choice for liver-affecting disorders.

Since LNPs delivered intravenously naturally accumulate in the liver, their effectiveness in targeting non-liver tissues is limited. LNPs can be modified to modulate their tropism by changing their molar percentage, administration site, or adding target-specific ligands in the cargo such as mRNA encoding tissue-targeting proteins, antibodies or aptamers (Loughrey and Dahlman, 2022). Adding anionic lipids successfully redirected liver-targeting LNP containing Cas9-mRNA to the spleen (Cheng et al., 2020); optimized LNPs for nebulized mRNA successfully delivered therapeutic mRNA to the lungs (Lokugamage et al., 2021); adding an mRNA encoding for vascular endothelial growth factor C increased lymphatic specificity (Szőke et al., 2021); and by intravitreal injection of LNP containing less PEG, therapeutic mRNA could arrive to retinal cells (Ryals et al., 2020).

##### 4.3.2.2 Virus-like particles (VLPs)

VLPs offer transient delivery by packaging mRNA, proteins or RNPs reducing the risk of off-target effects and viral integration (Banskota et al., 2022; Mangeot et al., 2019). VLPs are large enough to carry Cas9 and allow controlled delivery, minimizing off-target editing compared to AAVs and LVs (Banskota et al., 2022; Mangeot et al., 2019). They offer key benefits of both viral and non-viral delivery. Recent developments, like the PEG10-based SEND system, use endogenous proteins to improve efficiency and reduce immunogenicity (Segel et al., 2021), with potential for clinical applications if scaled up effectively.

### 4.4 Site of administration

Many intravenously injected vehicles can efficiently access some tissues, such as the liver, but cannot efficiently access others, such as the central nervous system (CNS), due to intrinsic biological barriers (e.g., the blood-brain barrier) (Daneman and Prat, 2015). Locally injecting vehicles into the CNS (e.g., via intrathecal injection) or eye (e.g., via subretinal injection) can circumvent biological barriers and enable access to certain important cell populations (Bottros and Christo, 2014; Peng et al., 2017).

## 5 Bench-to-bedside translation for base editing therapies for rare monogenic disorders

CRISPR-dependent base editing can revolutionize the treatment of rare monogenic disorders. The precision and efficiency of base editors provide hope for correcting pathogenic mutations that were once deemed untreatable. Yet, the transition from preclinical innovation to clinical implementation faces some hurdles.

Despite extensive base editor optimization, unintended off-target effects are still a primary concern. The observed targeting events with current base editors suggest high on-target and minimal off-target editing. However, base editors and the methods used for detecting their off-targets still require technical innovation, rigorous *in vivo* validation, and long-term studies for comprehensive assessment of safety profiles. The delivery of base editors also remains a bottleneck. Viral vectors, the most common delivery tools, have limited packaging capacities and immunogenic risks, whereas non-viral methods still require optimization for targeting specific tissues like the brain. Finally, base editing strategies can only be designed for diseases caused by transition mutations. This gap can be filled with newer, more versatile nickase-based gene editing technologies like prime editing (PE) or click editing (Ferreira da Silva et al., 2024). For instance, PE uses nickase Cas9 fused to reverse transcriptase (RT) and bound to a prime editing guide RNA (pegRNA) that can theoretically install precise insertions, deletions, and all twelve types of point mutations. Of note, advances in prime editors are directed to reduce their size, increase their efficiency, and reduce indel ratios, enhancing their therapeutic potential (Testa and Musunuru, 2023; Lee et al., 2023; Doman et al., 2023; Chen et al., 2021). Although still in the early stages of clinical application, PEs have been used *in vivo* in preclinical studies to correct pathogenic variants in liver diseases, such as tyrosinemia (Jiang et al., 2022) and phenylketonuria (Böck et al., 2022).

Beyond technical limitations, the therapeutic application of base editing must address complex regulatory and ethical considerations with evidence of efficacy, safety, and reproducibility before approval. This is particularly challenging for rare monogenic disorders: the lack of available treatments prompts shorter preclinical development times. Further, the small patient populations—often including primarily children—pose additional challenges to clinical trial design and implementation. The transition of base editing into clinical trials for a prevalent disease like heterozygous familial hypercholesterolemia establishes a strong precedent for rare disease therapeutics to follow. Optimized base editor configurations could be applied across multiple disorders, and learnings on safety and efficacy in broader patient populations (e.g., by determining optimal delivery vehicles or immunogenicity profiles) are critical to streamlining the progress of such therapies to the clinic, paving the way to design base editing therapeutics for rare monogenic disorders.

Finally, equitable access to these advanced therapies should remain a priority. Without proactive measures, the high costs associated with gene editing could exacerbate existing healthcare disparities, limiting the global impact of these innovations. Another key to advancing CRISPR-dependent base editing lies in collaborative efforts between academia, industry, and regulatory bodies. Public-private partnerships could accelerate the development of scalable manufacturing processes and standardized clinical protocols. By addressing the above challenges, the rare disorder field can navigate the complexities of translating CRISPR-dependent base editing from bench to bedside, paving the way for a new era of personalized medicine.

## References

[B1] Alves C. R. R. Ha L. L. Yaworski R. Sutton E. R. Lazzarotto C. R. Christie K. A. (2024). Optimization of base editors for the functional correction of SMN2 as a treatment for spinal muscular atrophy. Nat. Biomed. Eng. 8, 118–131. 10.1038/s41551-023-01132-z 38057426 PMC10922509

[B2] Anzalone A. V. Koblan L. W. Liu D. R. (2020). Genome editing with CRISPR-Cas nucleases, base editors, transposases and prime editors. Nat. Biotechnol. 38, 824–844. 10.1038/s41587-020-0561-9 32572269

[B3] Arbab M. Matuszek Z. Kray K. M. Du A. Newby G. A. Blatnik A. J. (2023). Base editing rescue of spinal muscular atrophy in cells and in mice. Science. 380, eadg6518. 10.1126/science.adg6518 36996170 PMC10270003

[B4] Arbab M. Shen M. W. Mok B. Wilson C. Matuszek Ż. Cassa C. A. (2020). Determinants of base editing outcomes from target library analysis and machine learning. Cell 182, 463–480.e30. 10.1016/j.cell.2020.05.037 32533916 PMC7384975

[B5] Arnaoutova I. Aratyn-Schaus Y. Zhang L. Packer M. S. Chen H. D. Lee C. (2024). Base-editing corrects metabolic abnormalities in a humanized mouse model for glycogen storage disease type-Ia. Nat. Commun. 15, 9729. 10.1038/s41467-024-54108-1 39523369 PMC11551175

[B6] Banskota S. Raguram A. Suh S. Du S. W. Davis J. R. Choi E. H. (2022). Engineered virus-like particles for efficient *in vivo* delivery of therapeutic proteins. Cell 185, 250–265.e16. 10.1016/j.cell.2021.12.021 35021064 PMC8809250

[B7] Böck D. Rothgangl T. Villiger L. Schmidheini L. Matsushita M. Mathis N. (2022). *In vivo* prime editing of a metabolic liver disease in mice. Sci. Transl. Med. 14, eabl9238. 10.1126/scitranslmed.abl9238 35294257 PMC7614134

[B8] Bose S. K. White B. M. Kashyap M. V. Dave A. De Bie F. R. Li H. (2021). *In utero* adenine base editing corrects multi-organ pathology in a lethal lysosomal storage disease. Nat. Commun. 12, 4291. 10.1038/s41467-021-24443-8 34257302 PMC8277817

[B9] Bottros M. M. Christo P. J. (2014). Current perspectives on intrathecal drug delivery. J. Pain Res. 7, 615–626. 10.2147/jpr.S37591 25395870 PMC4227625

[B10] Brooks D. L. Whittaker M. N. Said H. Dwivedi G. Qu P. Musunuru K. (2024). A base editing strategy using mRNA-LNPs for *in vivo* correction of the most frequent phenylketonuria variant. HGG Adv. 5, 100253. 10.1016/j.xhgg.2023.100253 37922902 PMC10800763

[B11] Chemello F. Chai A. C. Li H. Rodriguez-Caycedo C. Sanchez-Ortiz E. Atmanli A. (2021). Precise correction of Duchenne muscular dystrophy exon deletion mutations by base and prime editing. Sci. Adv. 7, eabg4910. 10.1126/sciadv.abg4910 33931459 PMC8087404

[B12] Chen P. J. Hussmann J. A. Yan J. Knipping F. Ravisankar P. Chen P. F. (2021). Enhanced prime editing systems by manipulating cellular determinants of editing outcomes. Cell 184, 5635–5652.e29. 10.1016/j.cell.2021.09.018 34653350 PMC8584034

[B13] Chen Y. Zhi S. Liu W. Wen J. Hu S. Cao T. (2020). Development of highly efficient dual‐AAV split adenosine base editor for *in vivo* gene therapy. Small Methods 4, 2000309. 10.1002/smtd.202000309

[B14] Cheng Q. Wei T. Farbiak L. Johnson L. T. Dilliard S. A. Siegwart D. J. (2020). Selective organ targeting (SORT) nanoparticles for tissue-specific mRNA delivery and CRISPR-Cas gene editing. Nat. Nanotechnol. 15, 313–320. 10.1038/s41565-020-0669-6 32251383 PMC7735425

[B15] Choi E. H. Suh S. Foik A. T. Leinonen H. Newby G. A. Gao X. D. (2022). *In vivo* base editing rescues cone photoreceptors in a mouse model of early-onset inherited retinal degeneration. Nat. Commun. 13, 1830. 10.1038/s41467-022-29490-3 35383196 PMC8983734

[B16] Collotta D. Bertocchi I. Chiapello E. Collino M. (2023). Antisense oligonucleotides: a novel Frontier in pharmacological strategy. Front. Pharmacol. 14, 1304342. 10.3389/fphar.2023.1304342 38044945 PMC10690781

[B17] Concordet J. P. Haeussler M. (2018). CRISPOR: intuitive guide selection for CRISPR/Cas9 genome editing experiments and screens. Nucleic Acids Res. 46, W242–W245. 10.1093/nar/gky354 29762716 PMC6030908

[B18] Cong L. Ran F. A. Cox D. Lin S. Barretto R. Habib N. (2013). Multiplex genome engineering using CRISPR/Cas systems. Science 339, 819–823. 10.1126/science.1231143 23287718 PMC3795411

[B19] Costa Verdera H. Kuranda K. Mingozzi F. (2020). AAV vector immunogenicity in humans: a long journey to successful gene transfer. Mol. Ther. 28, 723–746. 10.1016/j.ymthe.2019.12.010 31972133 PMC7054726

[B20] Cuella-Martin R. Hayward S. B. Fan X. Chen X. Huang J. W. Taglialatela A. (2021). Functional interrogation of DNA damage response variants with base editing screens. Cell 184, 1081–1097.e19. 10.1016/j.cell.2021.01.041 33606978 PMC8018281

[B21] Daneman R. Prat A. (2015). The blood-brain barrier. Cold Spring Harb. Perspect. Biol. 7, a020412. 10.1101/cshperspect.a020412 25561720 PMC4292164

[B22] Deneault E. (2024). Recent therapeutic gene editing applications to genetic disorders. Curr. Issues Mol. Biol. 46, 4147–4185. 10.3390/cimb46050255 38785523 PMC11119904

[B23] DeWeirdt P. C. McGee A. V. Zheng F. Nwolah I. Hegde M. Doench J. G. (2022). Accounting for small variations in the tracrRNA sequence improves sgRNA activity predictions for CRISPR screening. Nat. Commun. 13, 5255. 10.1038/s41467-022-33024-2 36068235 PMC9448816

[B24] Doench J. G. Fusi N. Sullender M. Hegde M. Vaimberg E. W. Donovan K. F. (2016). Optimized sgRNA design to maximize activity and minimize off-target effects of CRISPR-Cas9. Nat. Biotechnol. 34, 184–191. 10.1038/nbt.3437 26780180 PMC4744125

[B25] Doench J. G. Hartenian E. Graham D. B. Tothova Z. Hegde M. Smith I. (2014). Rational design of highly active sgRNAs for CRISPR-Cas9-mediated gene inactivation. Nat. Biotechnol. 32, 1262–1267. 10.1038/nbt.3026 25184501 PMC4262738

[B26] Doman J. L. Pandey S. Neugebauer M. E. An M. Davis J. R. Randolph P. B. (2023). Phage-assisted evolution and protein engineering yield compact, efficient prime editors. Cell 186, 3983–4002.e26. 10.1016/j.cell.2023.07.039 37657419 PMC10482982

[B27] Doman J. L. Raguram A. Newby G. A. Liu D. R. (2020). Evaluation and minimization of Cas9-independent off-target DNA editing by cytosine base editors. Nat. Biotechnol. 38, 620–628. 10.1038/s41587-020-0414-6 32042165 PMC7335424

[B28] Duan D. Goemans N. Takeda S. Mercuri E. Aartsma-Rus A. (2021). Duchenne muscular dystrophy. Nat. Rev. Dis. Prim. 7, 13. 10.1038/s41572-021-00248-3 33602943 PMC10557455

[B29] Eichler F. Duncan C. N. Musolino P. L. Lund T. C. Gupta A. O. De Oliveira S. (2024). Lentiviral gene therapy for cerebral adrenoleukodystrophy. N. Engl. J. Med. 391, 1302–1312. 10.1056/NEJMoa2400442 39383459 PMC12465018

[B30] FDA (2018). What is gene therapy? Available online at: https://www.fda.gov/vaccines-blood-biologics/cellular-gene-therapy-products/what-gene-therapy.

[B31] Ferreira da Silva J. Tou C. J. King E. M. Eller M. L. Rufino-Ramos D. Ma L. (2024). Click editing enables programmable genome writing using DNA polymerases and HUH endonucleases. Nat. Biotechnol. 10.1038/s41587-024-02324-x PMC11751136 39039307

[B32] Frangoul H. Locatelli F. Sharma A. Bhatia M. Mapara M. Molinari L. (2024). Exagamglogene autotemcel for severe sickle cell disease. N. Engl. J. Med. 390, 1649–1662. 10.1056/NEJMoa2309676 38661449

[B33] Gaudelli N. M. Komor A. C. Rees H. A. Packer M. S. Badran A. H. Bryson D. I. (2017). Programmable base editing of A·T to G·C in genomic DNA without DNA cleavage. Nature 551, 464–471. 10.1038/nature24644 29160308 PMC5726555

[B34] Grünewald J. Zhou R. Iyer S. Lareau C. A. Garcia S. P. Aryee M. J. (2019). CRISPR DNA base editors with reduced RNA off-target and self-editing activities. Nat. Biotechnol. 37, 1041–1048. 10.1038/s41587-019-0236-6 31477922 PMC6730565

[B35] Hanna R. E. Hegde M. Fagre C. R. DeWeirdt P. C. Sangree A. K. Szegletes Z. (2021). Massively parallel assessment of human variants with base editor screens. Cell 184, 1064–1080.e20. 10.1016/j.cell.2021.01.012 33606977

[B36] Hille F. Richter H. Wong S. P. Bratovič M. Ressel S. Charpentier E. (2018). The biology of CRISPR-cas: backward and forward. Cell 172, 1239–1259. 10.1016/j.cell.2017.11.032 29522745

[B37] Hmeljak J. Justice M. J. (2019). From gene to treatment: supporting rare disease translational research through model systems. Dis. Model Mech. 12, dmm039271. 10.1242/dmm.039271 30819728 PMC6398488

[B238] Horie T. Ono K. (2024). VERVE-101: a promising CRISPR-based gene editing therapy that reduces LDL-C and PCSK9 levels in HeFH patients. Eur. Heart J. Cardiovasc. Pharmacother. 10, 89–90. 10.1093/ehjcvp/pvad103 38142221

[B38] Hu J. H. Miller S. M. Geurts M. H. Tang W. Chen L. Sun N. (2018). Evolved Cas9 variants with broad PAM compatibility and high DNA specificity. Nature 556, 57–63. 10.1038/nature26155 29512652 PMC5951633

[B39] Hwang G. H. Park J. Lim K. Kim S. Yu J. Yu E. (2018). Web-based design and analysis tools for CRISPR base editing. BMC Bioinforma. 19, 542. 10.1186/s12859-018-2585-4 PMC6307267 30587106

[B40] Ibraheim R. Tai P. W. L. Mir A. Javeed N. Wang J. Rodríguez T. C. (2021). Self-inactivating, all-in-one AAV vectors for precision Cas9 genome editing via homology-directed repair *in vivo* . Nat. Commun. 12, 6267. 10.1038/s41467-021-26518-y 34725353 PMC8560862

[B41] Jiang F. Doudna J. A. (2017). CRISPR-Cas9 structures and mechanisms. Annu. Rev. Biophys. 46, 505–529. 10.1146/annurev-biophys-062215-010822 28375731

[B42] Jiang T. Henderson J. M. Coote K. Cheng Y. Valley H. C. Zhang X. O. (2020). Chemical modifications of adenine base editor mRNA and guide RNA expand its application scope. Nat. Commun. 11, 1979. 10.1038/s41467-020-15892-8 32332735 PMC7181807

[B43] Jiang T. Zhang X. O. Weng Z. Xue W. (2022). Deletion and replacement of long genomic sequences using prime editing. Nat. Biotechnol. 40, 227–234. 10.1038/s41587-021-01026-y 34650270 PMC8847310

[B44] Jin S. Zong Y. Gao Q. Zhu Z. Wang Y. Qin P. (2019). Cytosine, but not adenine, base editors induce genome-wide off-target mutations in rice. Science 364, 292–295. 10.1126/science.aaw7166 30819931

[B45] Jo D. H. Jang H. K. Cho C. S. Han J. H. Ryu G. Jung Y. (2023). Visual function restoration in a mouse model of Leber congenital amaurosis via therapeutic base editing. Mol. Ther. Nucleic Acids 31, 16–27. 10.1016/j.omtn.2022.11.021 36589710 PMC9792702

[B46] Kabra M. Shahi P. K. Wang Y. Sinha D. Spillane A. Newby G. A. (2023). Nonviral base editing of KCNJ13 mutation preserves vision in a model of inherited retinal channelopathy. J. Clin. Invest. 133, e171356. 10.1172/jci171356 37561581 PMC10541187

[B47] Kenjo E. Hozumi H. Makita Y. Iwabuchi K. A. Fujimoto N. Matsumoto S. (2021). Low immunogenicity of LNP allows repeated administrations of CRISPR-Cas9 mRNA into skeletal muscle in mice. Nat. Commun. 12, 7101. 10.1038/s41467-021-26714-w 34880218 PMC8654819

[B48] Kim J. Hu C. Moufawad El Achkar C. Black L. E. Douville J. Larson A. (2019). Patient-customized oligonucleotide therapy for a rare genetic disease. N. Engl. J. Med. 381, 1644–1652. 10.1056/NEJMoa1813279 31597037 PMC6961983

[B49] Kim J. Woo S. de Gusmao C. M. Zhao B. Chin D. H. DiDonato R. L. (2023). A framework for individualized splice-switching oligonucleotide therapy. Nature 619, 828–836. 10.1038/s41586-023-06277-0 37438524 PMC10371869

[B50] Kim Y. B. Komor A. C. Levy J. M. Packer M. S. Zhao K. T. Liu D. R. (2017). Increasing the genome-targeting scope and precision of base editing with engineered Cas9-cytidine deaminase fusions. Nat. Biotechnol. 35, 371–376. 10.1038/nbt.3803 28191901 PMC5388574

[B51] Kirschner J. Cathomen T. (2020). Gene therapy for monogenic inherited disorders. Dtsch. Arztebl Int. 117, 878–885. 10.3238/arztebl.2020.0878 33637169 PMC8045130

[B52] Kleinstiver B. P. Prew M. S. Tsai S. Q. Topkar V. V. Nguyen N. T. Zheng Z. (2015). Engineered CRISPR-Cas9 nucleases with altered PAM specificities. Nature 523, 481–485. 10.1038/nature14592 26098369 PMC4540238

[B53] Kleinstiver B. P. Sousa A. A. Walton R. T. Tak Y. E. Hsu J. Y. Clement K. (2019). Engineered CRISPR-Cas12a variants with increased activities and improved targeting ranges for gene, epigenetic and base editing. Nat. Biotechnol. 37, 276–282. 10.1038/s41587-018-0011-0 30742127 PMC6401248

[B54] Koblan L. W. Erdos M. R. Wilson C. Cabral W. A. Levy J. M. Xiong Z. M. (2021). *In vivo* base editing rescues Hutchinson-Gilford progeria syndrome in mice. Nature 589, 608–614. 10.1038/s41586-020-03086-7 33408413 PMC7872200

[B55] Komor A. C. Kim Y. B. Packer M. S. Zuris J. A. Liu D. R. (2016). Programmable editing of a target base in genomic DNA without double-stranded DNA cleavage. Nature 533, 420–424. 10.1038/nature17946 27096365 PMC4873371

[B56] Komor A. C. Zhao K. T. Packer M. S. Gaudelli N. M. Waterbury A. L. Koblan L. W. (2017). Improved base excision repair inhibition and bacteriophage Mu Gam protein yields C:G-to-T:A base editors with higher efficiency and product purity. Sci. Adv. 3, eaao4774. 10.1126/sciadv.aao4774 28875174 PMC5576876

[B57] Konstantakos V. Nentidis A. Krithara A. Paliouras G. (2022). CRISPR-Cas9 gRNA efficiency prediction: an overview of predictive tools and the role of deep learning. Nucleic Acids Res. 50, 3616–3637. 10.1093/nar/gkac192 35349718 PMC9023298

[B58] Kymäläinen H. Appelt J. U. Giordano F. A. Davies A. F. Ogilvie C. M. Ahmed S. G. (2014). Long-term episomal transgene expression from mitotically stable integration-deficient lentiviral vectors. Hum. Gene Ther. 25, 428–442. 10.1089/hum.2013.172 24483952 PMC4027990

[B59] Landrum M. J. Lee J. M. Benson M. Brown G. Chao C. Chitipiralla S. (2016). ClinVar: public archive of interpretations of clinically relevant variants. Nucleic Acids Res. 44, D862–D868. 10.1093/nar/gkv1222 26582918 PMC4702865

[B60] Lauffer M. C. van Roon-Mom W. Aartsma-Rus A. N = 1 Collaborative (2024). Possibilities and limitations of antisense oligonucleotide therapies for the treatment of monogenic disorders. Commun. Med. (Lond). 4, 6. 10.1038/s43856-023-00419-1 38182878 PMC10770028

[B61] Lazzarotto C. R. Katta V. Li Y. Urbina E. Lee G. Tsai S. Q. (2024). CHANGE-seq-BE enables simultaneously sensitive and unbiased *in vitro* profiling of base editor genome-wide activity. bioRxiv. 2024.03.28.586621. 10.1101/2024.03.28.586621 PMC13479990 41482541

[B62] Lee C. S. Bishop E. S. Zhang R. Yu X. Farina E. M. Yan S. (2017). Adenovirus-mediated gene delivery: potential applications for gene and cell-based therapies in the new era of personalized medicine. Genes Dis. 4, 43–63. 10.1016/j.gendis.2017.04.001 28944281 PMC5609467

[B63] Lee H. K. Smith H. E. Liu C. Willi M. Hennighausen L. (2020). Cytosine base editor 4 but not adenine base editor generates off-target mutations in mouse embryos. Commun. Biol. 3, 19. 10.1038/s42003-019-0745-3 31925293 PMC6952419

[B64] Lee J. Lim K. Kim A. Mok Y. G. Chung E. Cho S. I. (2023). Prime editing with genuine Cas9 nickases minimizes unwanted indels. Nat. Commun. 14, 1786. 10.1038/s41467-023-37507-8 36997524 PMC10063541

[B65] Lee S. H. Wu J. Im D. Hwang G. H. Jeong Y. K. Jiang H. (2024). Bystander base editing interferes with visual function restoration in Leber congenital amaurosis. bioRxiv. 2024.10.23.619839. 10.1101/2024.10.23.619839

[B66] Levy J. M. Yeh W. H. Pendse N. Davis J. R. Hennessey E. Butcher R. (2020). Cytosine and adenine base editing of the brain, liver, retina, heart and skeletal muscle of mice via adeno-associated viruses. Nat. Biomed. Eng. 4, 97–110. 10.1038/s41551-019-0501-5 31937940 PMC6980783

[B67] Li C. Georgakopoulou A. Mishra A. Gil S. Hawkins R. D. Yannaki E. (2021). *In vivo* HSPC gene therapy with base editors allows for efficient reactivation of fetal γ-globin in β-YAC mice. Blood Adv. 5, 1122–1135. 10.1182/bloodadvances.2020003702 33620431 PMC7903237

[B68] Li C. Georgakopoulou A. Newby G. A. Everette K. A. Nizamis E. Paschoudi K. (2022). *In vivo* base editing by a single i.v. vector injection for treatment of hemoglobinopathies. JCI Insight 7, e162939. 10.1172/jci.insight.162939 36006707 PMC9675455

[B69] Li G. Jin M. Li Z. Xiao Q. Lin J. Yang D. (2023). Mini-dCas13X-mediated RNA editing restores dystrophin expression in a humanized mouse model of Duchenne muscular dystrophy. J. Clin. Invest. 133, 133. 10.1172/jci162809 PMC9888377 36512423

[B70] Li X. Wang Y. Liu Y. Yang B. Wang X. Wei J. (2018). Base editing with a Cpf1-cytidine deaminase fusion. Nat. Biotechnol. 36, 324–327. 10.1038/nbt.4102 29553573

[B71] Lim C. K. W. Gapinske M. Brooks A. K. Woods W. S. Powell J. E. Zeballos C. M. (2020). Treatment of a mouse model of ALS by *in vivo* base editing. Mol. Ther. 28, 1177–1189. 10.1016/j.ymthe.2020.01.005 31991108 PMC7132599

[B72] Lin J. Jin M. Yang D. Li Z. Zhang Y. Xiao Q. (2024). Adenine base editing-mediated exon skipping restores dystrophin in humanized Duchenne mouse model. Nat. Commun. 15, 5927. 10.1038/s41467-024-50340-x 39009678 PMC11251194

[B73] Liu Z. Chen S. Shan H. Jia Y. Chen M. Song Y. (2020). Efficient base editing with high precision in rabbits using YFE-BE4max. Cell Death Dis. 11, 36. 10.1038/s41419-020-2244-3 31959743 PMC6971250

[B74] Lokugamage M. P. Vanover D. Beyersdorf J. Hatit M. Z. C. Rotolo L. Echeverri E. S. (2021). Optimization of lipid nanoparticles for the delivery of nebulized therapeutic mRNA to the lungs. Nat. Biomed. Eng. 5, 1059–1068. 10.1038/s41551-021-00786-x 34616046 PMC10197923

[B75] Loughrey D. Dahlman J. E. (2022). Non-liver mRNA delivery. Acc. Chem. Res. 55, 13–23. 10.1021/acs.accounts.1c00601 34859663

[B76] Ma Y. Zhang J. Yin W. Zhang Z. Song Y. Chang X. (2016). Targeted AID-mediated mutagenesis (TAM) enables efficient genomic diversification in mammalian cells. Nat. Methods 13, 1029–1035. 10.1038/nmeth.4027 27723754

[B77] Mali P. Yang L. Esvelt K. M. Aach J. Guell M. DiCarlo J. E. (2013). RNA-guided human genome engineering via Cas9. Science 339, 823–826. 10.1126/science.1232033 23287722 PMC3712628

[B78] Mangeot P. E. Risson V. Fusil F. Marnef A. Laurent E. Blin J. (2019). Genome editing in primary cells and *in vivo* using viral-derived Nanoblades loaded with Cas9-sgRNA ribonucleoproteins. Nat. Commun. 10, 45. 10.1038/s41467-018-07845-z 30604748 PMC6318322

[B79] Miller S. M. Wang T. Randolph P. B. Arbab M. Shen M. W. Huang T. P. (2020). Continuous evolution of SpCas9 variants compatible with non-G PAMs. Nat. Biotechnol. 38, 471–481. 10.1038/s41587-020-0412-8 32042170 PMC7145744

[B80] Milone M. C. O'Doherty U. (2018). Clinical use of lentiviral vectors. Leukemia 32, 1529–1541. 10.1038/s41375-018-0106-0 29654266 PMC6035154

[B81] Musunuru K. Chadwick A. C. Mizoguchi T. Garcia S. P. DeNizio J. E. Reiss C. W. (2021). *In vivo* CRISPR base editing of PCSK9 durably lowers cholesterol in primates. Nature 593, 429–434. 10.1038/s41586-021-03534-y 34012082

[B82] National Institutes of Health (2023). The promise of precision medicine. Rare diseases. Available online at: https://www.nih.gov/about-nih/what-we-do/nih-turning-discovery-into-health/promise-precision-medicine/rare-diseases.

[B83] Nishimasu H. Ran F. A. Hsu P. D. Konermann S. Shehata S. I. Dohmae N. (2014). Crystal structure of Cas9 in complex with guide RNA and target DNA. Cell 156, 935–949. 10.1016/j.cell.2014.02.001 24529477 PMC4139937

[B84] Nishimasu H. Shi X. Ishiguro S. Gao L. Hirano S. Okazaki S. (2018). Engineered CRISPR-Cas9 nuclease with expanded targeting space. Science 361, 1259–1262. 10.1126/science.aas9129 30166441 PMC6368452

[B85] Packer M. S. Chowdhary V. Lung G. Cheng L. I. Aratyn-Schaus Y. Leboeuf D. (2022). Evaluation of cytosine base editing and adenine base editing as a potential treatment for alpha-1 antitrypsin deficiency. Mol. Ther. 30, 1396–1406. 10.1016/j.ymthe.2022.01.040 35121111 PMC9077367

[B86] Padda I. S. Mahtani A. U. Patel P. Parmar M. (2025). “Small interfering RNA (siRNA) therapy,” in StatPearls (Treasure Island (FL): StatPearls Publishing). 35593797

[B87] Palanki R. Bose S. K. Dave A. White B. M. Berkowitz C. Luks V. (2023). Ionizable lipid nanoparticles for therapeutic base editing of congenital brain disease. ACS Nano 17, 13594–13610. 10.1021/acsnano.3c02268 37458484 PMC11025390

[B88] Peng Y. Tang L. Zhou Y. (2017). Subretinal injection: a review on the novel route of therapeutic delivery for vitreoretinal diseases. Ophthalmic Res. 58, 217–226. 10.1159/000479157 28858866

[B89] Petrich J. Marchese D. Jenkins C. Storey M. Blind J. (2020). Gene replacement therapy: a primer for the health-system pharmacist. J. Pharm. Pract. 33, 846–855. 10.1177/0897190019854962 31248331 PMC7675776

[B90] Philippidis A. (2024). Verve pauses enrollment in base editing trial after adverse events. Hum. Gene Ther. 35, 313–316. 10.1089/hum.2024.28412.bfs 38781422

[B91] Qi M. Ma S. Liu J. Liu X. Wei J. Lu W. J. (2024). *In vivo* base editing of Scn5a rescues type 3 long QT syndrome in mice. Circulation 149, 317–329. 10.1161/circulationaha.123.065624 37965733

[B92] Radany E. H. Dornfeld K. J. Sanderson R. J. Savage M. K. Majumdar A. Seidman M. M. (2000). Increased spontaneous mutation frequency in human cells expressing the phage PBS2-encoded inhibitor of uracil-DNA glycosylase. Mutat. Res. 461, 41–58. 10.1016/s0921-8777(00)00040-9 10980411

[B93] Raguram A. Banskota S. Liu D. R. (2022). Therapeutic *in vivo* delivery of gene editing agents. Cell 185, 2806–2827. 10.1016/j.cell.2022.03.045 35798006 PMC9454337

[B94] Rees H. A. Komor A. C. Yeh W. H. Caetano-Lopes J. Warman M. Edge A. S. B. (2017). Improving the DNA specificity and applicability of base editing through protein engineering and protein delivery. Nat. Commun. 8, 15790. 10.1038/ncomms15790 28585549 PMC5467206

[B95] Rees H. A. Wilson C. Doman J. L. Liu D. R. (2019). Analysis and minimization of cellular RNA editing by DNA adenine base editors. Sci. Adv. 5, eaax5717. 10.1126/sciadv.aax5717 31086823 PMC6506237

[B96] Richter M. F. Zhao K. T. Eton E. Lapinaite A. Newby G. A. Thuronyi B. W. (2020). Phage-assisted evolution of an adenine base editor with improved Cas domain compatibility and activity. Nat. Biotechnol. 38, 883–891. 10.1038/s41587-020-0453-z 32433547 PMC7357821

[B97] Rothgangl T. Dennis M. K. Lin P. J. C. Oka R. Witzigmann D. Villiger L. (2021). *In vivo* adenine base editing of PCSK9 in macaques reduces LDL cholesterol levels. Nat. Biotechnol. 39, 949–957. 10.1038/s41587-021-00933-4 34012094 PMC8352781

[B98] Ryals R. C. Patel S. Acosta C. McKinney M. Pennesi M. E. Sahay G. (2020). The effects of PEGylation on LNP based mRNA delivery to the eye. PLoS One 15, e0241006. 10.1371/journal.pone.0241006 33119640 PMC7595320

[B99] Ryu S. M. Koo T. Kim K. Lim K. Baek G. Kim S. T. (2018). Adenine base editing in mouse embryos and an adult mouse model of Duchenne muscular dystrophy. Nat. Biotechnol. 36, 536–539. 10.1038/nbt.4148 29702637

[B100] Sanson K. R. Hanna R. E. Hegde M. Donovan K. F. Strand C. Sullender M. E. (2018). Optimized libraries for CRISPR-Cas9 genetic screens with multiple modalities. Nat. Commun. 9, 5416. 10.1038/s41467-018-07901-8 30575746 PMC6303322

[B101] Scott T. Urak R. Soemardy C. Morris K. V. (2019). Improved Cas9 activity by specific modifications of the tracrRNA. Sci. Rep. 9, 16104. 10.1038/s41598-019-52616-5 31695072 PMC6834579

[B102] Segel M. Lash B. Song J. Ladha A. Liu C. C. Jin X. (2021). Mammalian retrovirus-like protein PEG10 packages its own mRNA and can be pseudotyped for mRNA delivery. Science. 373, 882–889. 10.1126/science.abg6155 34413232 PMC8431961

[B103] Skeens E. Sinha S. Ahsan M. D'Ordine A. M. Jogl G. Palermo G. (2024). High-fidelity, hyper-accurate, and evolved mutants rewire atomic-level communication in CRISPR-Cas9. Sci. Adv. 10, eadl1045. 10.1126/sciadv.adl1045 38446895 PMC10917355

[B104] Song C. Q. Jiang T. Richter M. Rhym L. H. Koblan L. W. Zafra M. P. (2020). Adenine base editing in an adult mouse model of tyrosinaemia. Nat. Biomed. Eng. 4, 125–130. 10.1038/s41551-019-0357-8 31740768 PMC6986236

[B105] Song Z. Tao Y. Liu Y. Li J. (2024). Advances in delivery systems for CRISPR/Cas-mediated cancer treatment: a focus on viral vectors and extracellular vesicles. Front. Immunol. 15, 1444437. 10.3389/fimmu.2024.1444437 39281673 PMC11392784

[B106] Sternberg S. H. Redding S. Jinek M. Greene E. C. Doudna J. A. (2014). DNA interrogation by the CRISPR RNA-guided endonuclease Cas9. Nature 507, 62–67. 10.1038/nature13011 24476820 PMC4106473

[B107] Su J. Jin X. She K. Liu Y. Song L. Zhao Q. (2023). *In vivo* adenine base editing corrects newborn murine model of Hurler syndrome. Mol. Biomed. 4, 6. 10.1186/s43556-023-00120-8 36813914 PMC9947215

[B108] Suh S. Choi E. H. Leinonen H. Foik A. T. Newby G. A. Yeh W. H. (2021). Restoration of visual function in adult mice with an inherited retinal disease via adenine base editing. Nat. Biomed. Eng. 5, 169–178. 10.1038/s41551-020-00632-6 33077938 PMC7885272

[B109] Szőke D. Kovács G. Kemecsei É. Bálint L. Szoták-Ajtay K. Aradi P. (2021). Nucleoside-modified VEGFC mRNA induces organ-specific lymphatic growth and reverses experimental lymphedema. Nat. Commun. 12, 3460. 10.1038/s41467-021-23546-6 34103491 PMC8187400

[B110] Testa L. C. Musunuru K. (2023). Base editing and prime editing: potential therapeutic options for rare and common diseases. BioDrugs 37, 453–462. 10.1007/s40259-023-00610-9 37314680

[B111] The Lancet Global H. (2024). The landscape for rare diseases in 2024. Lancet Glob. Health 12, e341. 10.1016/S2214-109X(24)00056-1 38365397

[B112] Tsai S. Q. Nguyen N. T. Malagon-Lopez J. Topkar V. V. Aryee M. J. Joung J. K. (2017). CIRCLE-seq: a highly sensitive *in vitro* screen for genome-wide CRISPR-Cas9 nuclease off-targets. Nat. Methods 14, 607–614. 10.1038/nmeth.4278 28459458 PMC5924695

[B113] Tsai S. Q. Zheng Z. Nguyen N. T. Liebers M. Topkar V. V. Thapar V. (2015). GUIDE-seq enables genome-wide profiling of off-target cleavage by CRISPR-Cas nucleases. Nat. Biotechnol. 33, 187–197. 10.1038/nbt.3117 25513782 PMC4320685

[B114] Vafai S. Karsten V. Jensen C. Falzone R. Lister T. Stolz L. (2024). Abstract 4139206: design of Heart-2: a phase 1b clinical trial of VERVE-102, an *in vivo* base editing medicine delivered by a GalNAc-LNP and targeting *PCSK9* to durably lower LDL cholesterol. Circulation 150, A4139206. 10.1161/circ.150.suppl_1.4139206

[B116] Villiger L. Grisch-Chan H. M. Lindsay H. Ringnalda F. Pogliano C. B. Allegri G. (2018). Treatment of a metabolic liver disease by *in vivo* genome base editing in adult mice. Nat. Med. 24, 1519–1525. 10.1038/s41591-018-0209-1 30297904

[B117] Villiger L. Rothgangl T. Witzigmann D. Oka R. Lin P. J. C. Qi W. (2021). *In vivo* cytidine base editing of hepatocytes without detectable off-target mutations in RNA and DNA. Nat. Biomed. Eng. 5, 179–189. 10.1038/s41551-020-00671-z 33495639 PMC7610981

[B118] Vinjamuri B. P. Pan J. Peng P. (2024). A review on commercial oligonucleotide drug products. J. Pharm. Sci. 113, 1749–1768. 10.1016/j.xphs.2024.04.021 38679232

[B119] Walton R. T. Christie K. A. Whittaker M. N. Kleinstiver B. P. (2020). Unconstrained genome targeting with near-PAMless engineered CRISPR-Cas9 variants. Science 368, 290–296. 10.1126/science.aba8853 32217751 PMC7297043

[B120] Wang D. Mou H. Li S. Li Y. Hough S. Tran K. (2015). Adenovirus-mediated somatic genome editing of pten by CRISPR/Cas9 in mouse liver in spite of Cas9-specific immune responses. Hum. Gene Ther. 26, 432–442. 10.1089/hum.2015.087 26086867 PMC4509492

[B121] Wang D. Zhang Y. Zhang J. Zhao J. (2024). Advances in base editing: a focus on base transversions. Mutat. Res. Rev. Mutat. Res. 794, 108515. 10.1016/j.mrrev.2024.108515 39454989

[B122] Wang L. Xue W. Yan L. Li X. Wei J. Chen M. (2017). Enhanced base editing by co-expression of free uracil DNA glycosylase inhibitor. Cell Res. 27, 1289–1292. 10.1038/cr.2017.111 28849781 PMC5630677

[B123] Wang Y. Hu L. F. Zhou T. J. Qi L. Y. Xing L. Lee J. (2021). Gene therapy strategies for rare monogenic disorders with nuclear or mitochondrial gene mutations. Biomaterials 277, 121108. 10.1016/j.biomaterials.2021.121108 34478929

[B124] Whisenant D. Lim K. Revêchon G. Yao H. Bergo M. O. Machtel P. (2022). Transient expression of an adenine base editor corrects the Hutchinson-Gilford progeria syndrome mutation and improves the skin phenotype in mice. Nat. Commun. 13, 3068. 10.1038/s41467-022-30800-y 35654881 PMC9163128

[B125] Wienert B. Wyman S. K. Richardson C. D. Yeh C. D. Akcakaya P. Porritt M. J. (2019). Unbiased detection of CRISPR off-targets *in vivo* using DISCOVER-Seq. Science 364, 286–289. 10.1126/science.aav9023 31000663 PMC6589096

[B126] Wood R. D. (1996). DNA repair in eukaryotes. Annu. Rev. Biochem. 65, 135–167. 10.1146/annurev.bi.65.070196.001031 8811177

[B127] Wu Y. Xu W. Wang F. Zhao S. Feng F. Song J. (2019). Increasing cytosine base editing scope and efficiency with engineered Cas9-PmCDA1 fusions and the modified sgRNA in rice. Front. Genet. 10, 379. 10.3389/fgene.2019.00379 31134125 PMC6512751

[B128] Xu Y. Li Z. (2020). CRISPR-Cas systems: overview, innovations and applications in human disease research and gene therapy. Comput. Struct. Biotechnol. J. 18, 2401–2415. 10.1016/j.csbj.2020.08.031 33005303 PMC7508700

[B129] Xue Y. Tao Y. Wang X. Wang X. Shu Y. Liu Y. (2023). RNA base editing therapy cures hearing loss induced by OTOF gene mutation. Mol. Ther. 31, 3520–3530. 10.1016/j.ymthe.2023.10.019 37915172 PMC10727966

[B130] Yan Q. Li D. Jia S. Yang J. Ma J. (2024). Novel gene-based therapeutic approaches for the management of hepatic complications in diabetes: reviewing recent advances. J. Diabetes Complicat. 38, 108688. 10.1016/j.jdiacomp.2024.108688 38281457

[B131] Yang L. Huo Y. Wang M. Zhang D. Zhang T. Wu H. (2024). Engineering APOBEC3A deaminase for highly accurate and efficient base editing. Nat. Chem. Biol. 20, 1176–1187. 10.1038/s41589-024-01595-4 38553609

[B132] Yeh W. H. Shubina-Oleinik O. Levy J. M. Pan B. Newby G. A. Wornow M. (2020). *In vivo* base editing restores sensory transduction and transiently improves auditory function in a mouse model of recessive deafness. Sci. Transl. Med. 12, eaay9101. 10.1126/scitranslmed.aay9101 32493795 PMC8167884

[B133] Yin S. Gao L. Sun X. Zhang M. Gao H. Chen X. (2025). Amelioration of metabolic and behavioral defects through base editing in the Pah(R408W) phenylketonuria mouse model. Mol. Ther. 33, 119–132. 10.1016/j.ymthe.2024.11.032 39600089 PMC11764323

[B134] Yu Y. Leete T. C. Born D. A. Young L. Barrera L. A. Lee S. J. (2020). Cytosine base editors with minimized unguided DNA and RNA off-target events and high on-target activity. Nat. Commun. 11, 2052. 10.1038/s41467-020-15887-5 32345976 PMC7189382

[B135] Yuan J. Ma Y. Huang T. Chen Y. Peng Y. Li B. (2018). Genetic modulation of RNA splicing with a CRISPR-guided cytidine deaminase. Mol. Cell 72, 380–394.e7. 10.1016/j.molcel.2018.09.002 30293782

[B136] Zhang Y. Wu Z. Y. (2024). Gene therapy for monogenic disorders: challenges, strategies, and perspectives. J. Genet. Genomics 51, 133–143. 10.1016/j.jgg.2023.08.001 37586590

[B137] Zhao D. Li J. Li S. Xin X. Hu M. Price M. A. (2021). Glycosylase base editors enable C-to-A and C-to-G base changes. Nat. Biotechnol. 39, 35–40. 10.1038/s41587-020-0592-2 32690970

[B138] Zhou C. Sun Y. Yan R. Liu Y. Zuo E. Gu C. (2019). Off-target RNA mutation induced by DNA base editing and its elimination by mutagenesis. Nature 571, 275–278. 10.1038/s41586-019-1314-0 31181567

[B139] Zhou L. Su J. Long J. Tao R. Tang W. Qin F. (2022). A universal strategy for AAV delivery of base editors to correct genetic point mutations in neonatal PKU mice. Mol. Ther. Methods Clin. Dev. 24, 230–240. 10.1016/j.omtm.2022.01.001 35141352 PMC8803597

[B140] Zuo E. Sun Y. Wei W. Yuan T. Ying W. Sun H. (2019). Cytosine base editor generates substantial off-target single-nucleotide variants in mouse embryos. Science. 364, 289–292. 10.1126/science.aav9973 30819928 PMC7301308

